# Enhancing performance of gravitational water vortex turbines through airfoil design and basin parameters

**DOI:** 10.1038/s41598-026-56885-9

**Published:** 2026-06-18

**Authors:** Miral Michel, Abdelmenaim Hatim Alaktaa, Khaled Elsherbiny, Ahmed Mehanna, Ahmed S. Shehata

**Affiliations:** Marine and Offshore Engineering Department, College of Engineering and Technology, Arab Academy for Science Technology and Maritime Transport, Alexandria, Egypt

**Keywords:** Whirlpool hydropower generator (WHG), Low-head hydropower, Conical vortex basin, Computational fluid dynamics (CFD), Airfoil spacing, Basin airfoil, Airfoil sizing., Energy science and technology, Engineering

## Abstract

Gravitational water vortex turbines (GWVTs) have emerged as promising hydrokinetic technology for energy extraction in riverine systems characterized by low flow velocity and ultra-low head, where conventional hydropower solutions are not feasible. In this study, a three-dimensional computational fluid dynamics (CFD) model is developed to investigate turbine performance within a conical basin configuration. The numerical framework, validated against published reference data, is used to systematically evaluate the influence of airfoil axial spacing, chord sizing, and airfoil profile, on vortex structure and power coefficient. The results demonstrate that basin-induced flow control plays a critical role in enhancing tangential momentum transfer to the rotor. Among the investigated cases, a configuration employing a NACA0024 ducted airfoil with a chord size of 35 mm and an axial spacing of 50 mm yielded the highest power coefficient of 0.419 compared to the baseline basin power coefficient of 0.313 and reached a maximum value of 0.736 at higher inlet velocities. Performance analysis based on tip speed ratio (TSR) further identified an optimal low-TSR operating range consistent with gravitational water vortex turbine characteristics. Compared with previous GWVT studies that focus on turbine geometry, the present work highlights the effectiveness of basin wall airfoil design as an impactful strategy for performance enhancement in low-head hydrokinetic applications.

## Introduction

Hydropower is expanding to provide green energy from small scale to large scale. The form of which is hydrokinetic energy that can generate energy from rivers, channels, and even irrigation systems. That type of energy extraction is suitable for areas with low water head and low water velocity. These hydrokinetic systems are applicable and already used at several locations worldwide with proven technology. Hydrokinetic energy systems also improve environmental sustainability, making it an interesting topic for researchers. Furthermore, Egypt has a large riverine system that is regularly used at rural areas to provide energy on a small scale. Whirlpool hydropower generators (WHG) require low water heads with flowing water where the gravitational force drives the flowing water to generate an artificial whirlpool vortex^1^ by a specific system’s geometry that directs the water flow motion into a conical pass^2^. Hydropower plants are categorized according to the potential energy from Pico, Micro, Mini, Small, to Large scales; ranging from 0.005, 0.1, 1, 10, to larger than 10 MWs^3^.

The WHG is superior to other technologies due to its potential to rotate by low-flow conditions under ultra-low water head range of 0.7–2 m^4^. The main concept relies on the artificially induced vortex, which is generated in a cylindrical basin with an orifice at its bottom. Once the flowing water enters and passes through a straight channel as an upstream flow affected by the earth gravity, the vortex is initiated. As the flow is directed tangentially to the cylindrical basin, vortex forms through the surface of the water caused by the Coriolis force. The generated vortex starts to move directly to the center of the basin as preparation to be discharged out of the basin orifice under gravitational force.

The vortex gradually intensifies its power from gravity and increases its size and velocity while it moves toward the orifice, causing the water rotational speed to go up. As the velocity increases, the pressure decreases below the atmospheric pressure and creates pressure difference. The atmospheric air enters in the basin at the center and forms an air core under the force of the pressure difference. With the variation of the pressure and the water velocity, the radius of the air core gradually decreases while it goes away from the surface to the bottom^5^. The discharged water drained again into the flowing stream; the cycle goes on. Then, by using a mechanical rotating mechanism as turbines, this vortex energy can be extracted from the flow^6^. A vertical-axis turbine is placed in the center of the water vortex and rotates coaxially with it to harness kinetic energy which has a small orifice at the bottom. The rotating water pushes equally over all the blades at the same time^7^. Tangential velocity provides the maximum kinetic energy for the turbine compared to axial and radial velocities [15]. The interaction between the rotor and the vortex itself results in losses^2,9^. A guide plate before the inlet to vortex chamber achieves the induced circulation [9]. Higher inlet flow rate gives the better efficiency of whirlpool hydropower generator.

Several research methods tackled hydrokinetic energy systems, whereas many of them have investigated the improvements of the concept. Jeon et al.^10^, investigated the baffle plates installed on the highest and lowest points of a Savonius wind turbine blades, which increased the efficiency by 36%. S. Dhakal et al.^11^, experimentally evaluated the optimal location to increase the turbine efficiency near to the basin bottom. Gautam et al.^12^, numerically simulated then experimented a conical basin with coupling multiple turbine runners on one shaft, in basin setup as S. Dhakal et al.^13^, which has increased the efficiency by 6%. Power et al.^14^, experimentally investigated a paddle-type runner in the cylindrical basin, while varying blade size, blade number, inlet flow rate, and inlet height. The results showed that efficiency changes with changing the blade area and blade number.

Wichian and Suntivarakorn^15^ influenced by Jeon et al.^10^, numerically investigated turbine baffle plates on a whirlpool hydropower turbine and found an increase in torque and efficiency by 10.25% and 4.12% respectively while using a turbine with 5 blades and 50% baffle plates as the optimal design. In 2017, Kueh et al.^16^, proved that the curved blades are better than the straight blades. Further, R. Dhakal and Khanal^17^ numerically investigated the importance of the blade angle which revealed that an angle of 19° was the optimal between the range studied from 15° to 25°.

Sean Mulligan et al.^18^, investigated several geometric parameters with different operating conditions, and compared with Mulligan et al.’s^19^ model. The results revealed similar trends and a complex relationship between the geometric parameters and volumetric flow rate which affects the circulation number and geometry factor. Large vortex formations occur and could be affected by turbulent flow, therefore, Ullah et al.^20^, analytically and experimentally investigated the performance of a multi-stage gravitational water vortex turbine in a conical basin. These runners are rotating separately through telescopic shaft arrangement. The results showed an improvement in the overall performance compared to a single-stage runner. Furthermore, the system had a small investment cost with simple operations and short payback time.

Saleem et al.^6^, mathematically and experimentally studied different performance parameters of a gravitational water vortex turbine which showed that vortex height and shape with fully developed air core are crucial for the performance. Ullah et al.^21^, experimentally investigated the crucial design parameters and the performance parameters of a vortex Savonius rotor. The results showed that the multi-stage runner generates more power than single stage runners. Alzamora Guzmán and Glasscock^22^ analytically modeled a strong free-surface of a water vortex. The model overcomes some limitations of the design such as the volumetric flow rate and provides precise calculations of the resultant velocity vectors.

S. Dhakal et al.^11^, investigated the geometric parameters affecting vortex formation of a conical basin. The results revealed that basin geometry plays a role in changing the vortex velocity as well as all parameters. Therefore, the generated swirl of the V-shaped base is effective in generating a hydrodynamic movement on the attached middle runner to generate power. The increased inlet current velocity that is directed by the inlet notch increases the swirl velocity by turn. The maximum swirl velocity is achieved by an inlet current velocity of 3.1 m/s. Also, the maximum velocity inside the basin was observed at the basin’s side and the basin’s outlet, while the velocity at the middle is very low.

Despite the extensive research on GWVT, several limitations remain in existing literature as most studies focused on isolated turbine parameters such as blade geometry, blade number, or runner position, while the influence of basin design on vortex strength is often fixed. Furthermore, many numerical and experimental studies are conducted under idealized boundary conditions that do not fully reflect the variability of real riverine and canal environments. While average river velocities are often relatively low, localized higher velocities can occur in constrained channels, irrigation canals, and engineered flow sections, which are relevant deployment scenarios for gravitational water vortex turbines. Accordingly, the inlet velocities considered in this study (1.6–3.1 m/s) represent upper-bound operational scenarios intended to assess performance trends and optimization potential of gravitational water vortex turbines.

This study aims to address these gaps by numerically studying the influence of basin structure geometry on vortex formation and turbine performance using computational fluid dynamics (CFD). This study focuses on optimizing basin design parameters under boundary conditions representative of riverine environments to provide design modifications suitable for real-world low-head hydrokinetic applications.

The scope of this study is limited to the numerical investigation of basin wall design parameters and their influence on vortex formation, flow structure, and the resulting rotor performance in a gravitational water vortex turbine system. The analyzed variables include basin geometry, inlet configuration, and flow boundary conditions representing riverine environments. Turbine blade geometry and mechanical efficiency are not optimized in this study and are treated as fixed parameters. These limitations are acknowledged and will be addressed in future experimental studies.

## Methodology

**Fig. 1:**
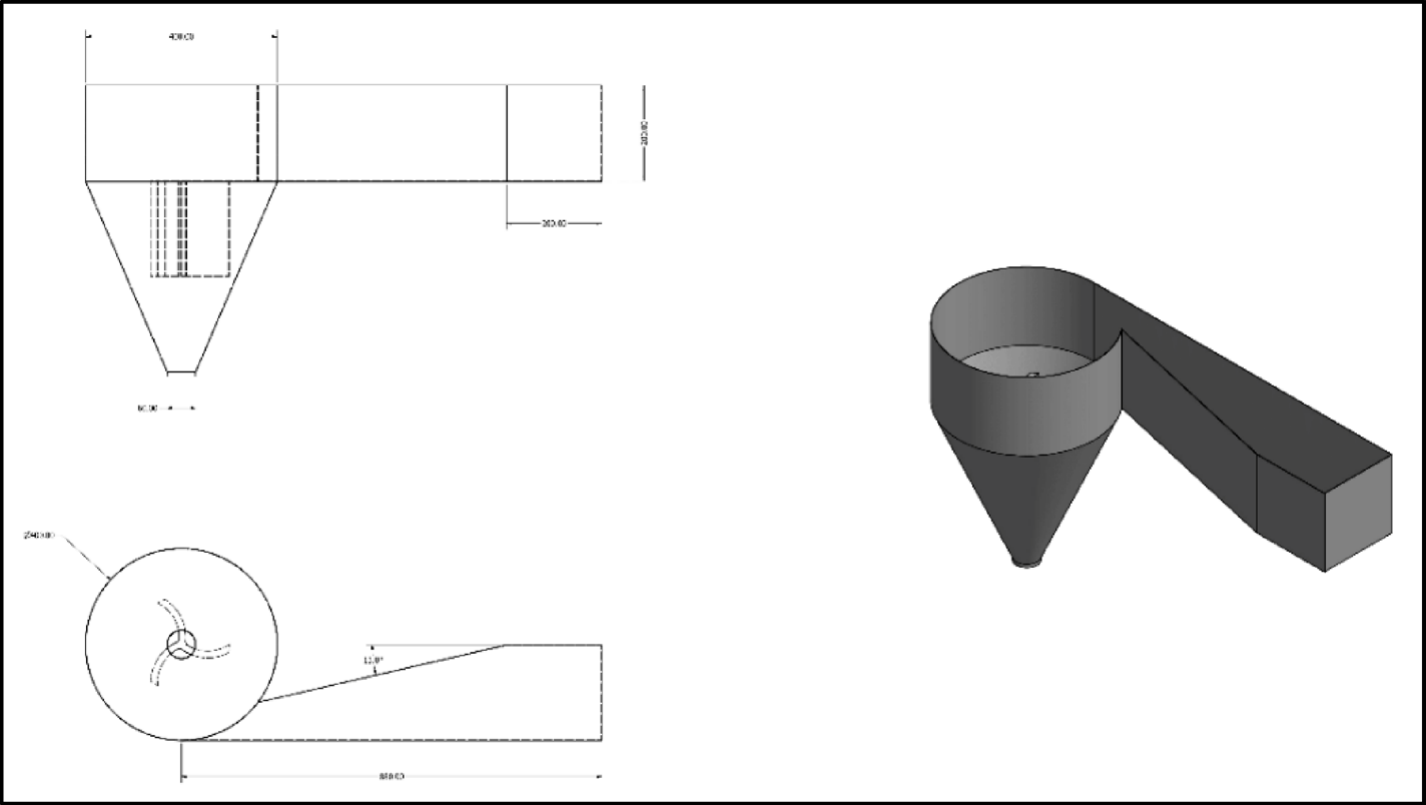
The geometry and dimensions of the V-shaped model basin employed with the runner.

Numerical analyses were carried out to examine the improvement of the current turbine by studying and measuring hydrodynamic coefficients. Numerical analysis was implemented using ANSYS Fluent software 2022 R1. The basin model was shaped as a conical drop chamber, which allows the water to reach the conical basin tangentially through a canal having a notch of 13°, at the side connecting the conical basin inlet as shown in Fig. 1.

**Fig. 2:**
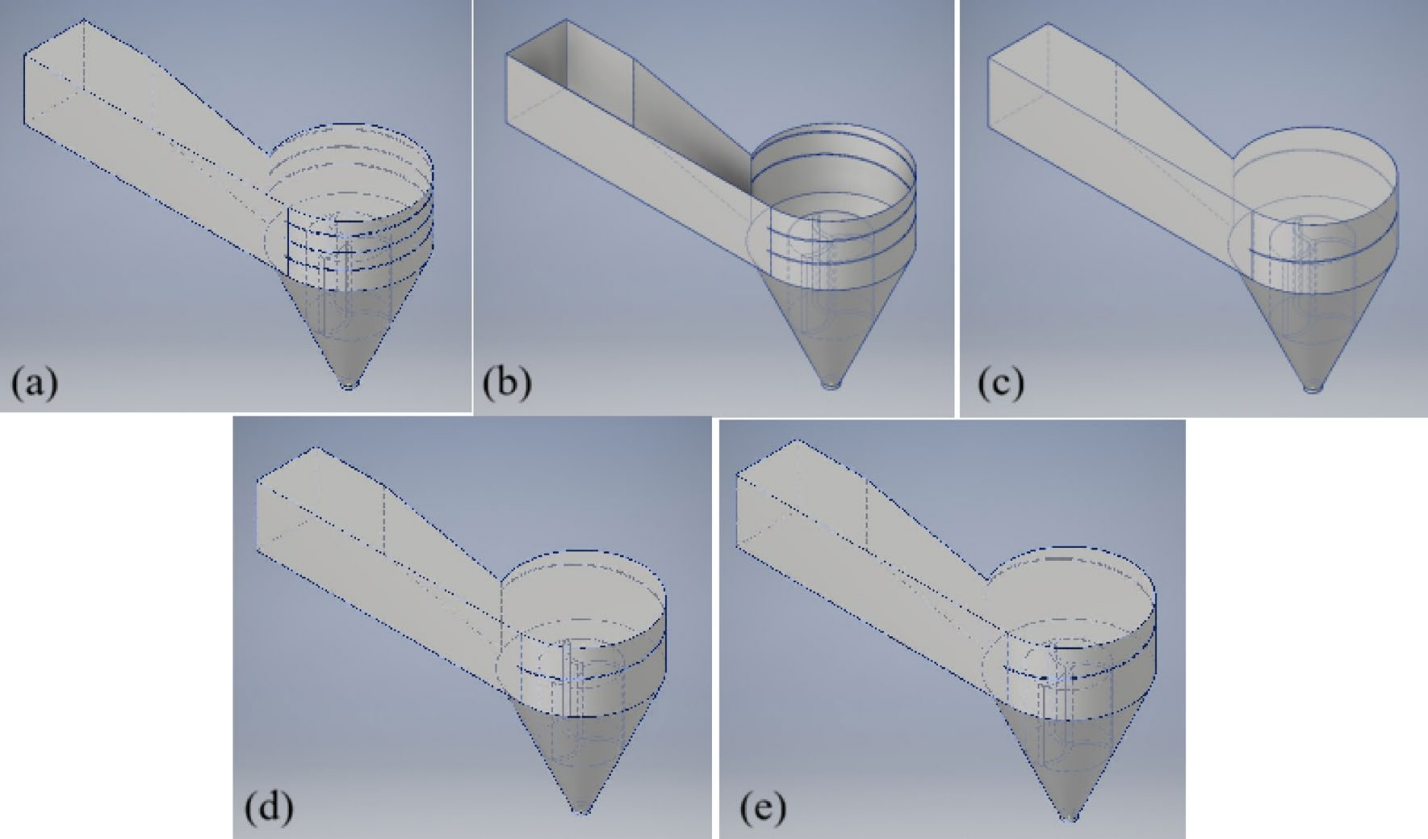
Varying airfoils’ axial spacing of 50 mm, 65 mm, 80 mm, 95 mm, and 110 mm for (**a**), (**b**), (**c**), (**d**), and (**e**) respectively.

This fixed design allowed the performance of a parametric analysis for the basin side wall with different helical adjustments. Three adjustment methods were used to improve the turbine hydrodynamics by enhancing the generated water vortex. The first method was varying the airfoils’ axial spacing as follows: 50 mm, 65 mm, 80 mm, 95 mm, and 110 mm, as shown in Fig. 2.

**Fig. 3:**
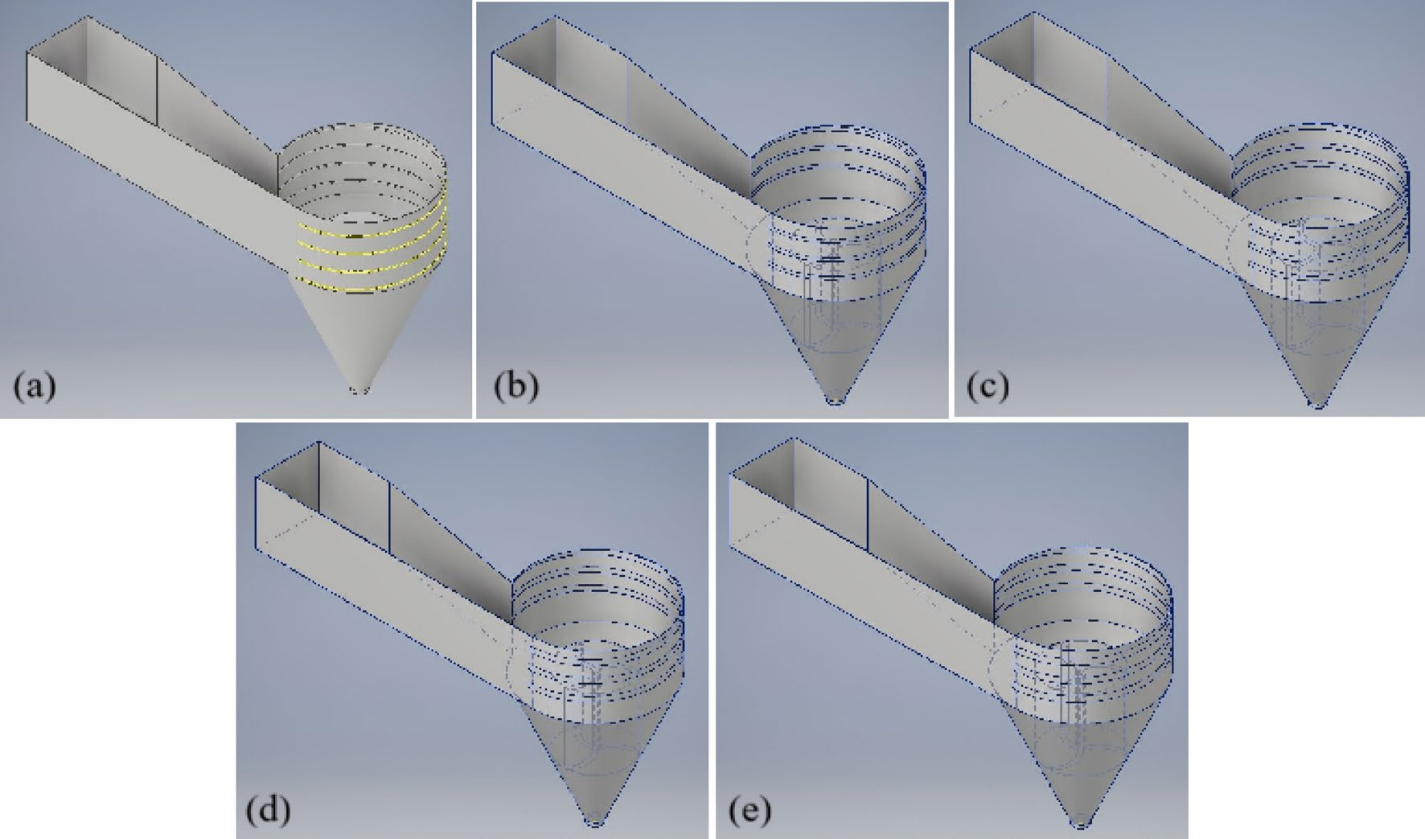
Varying sizing of the ducted airfoil of 35 mm, 50 mm, 65 mm, 80 mm, and 95 mm for (**a**), (**b**), (**c**), (**d**), and (**e**) respectively.

Secondly, the ducted airfoil sizing varied as follows: 35 mm, 50 mm, 65 mm, 80 mm, and 95 mm, as shown in Fig. 3.

**Fig. 4:**
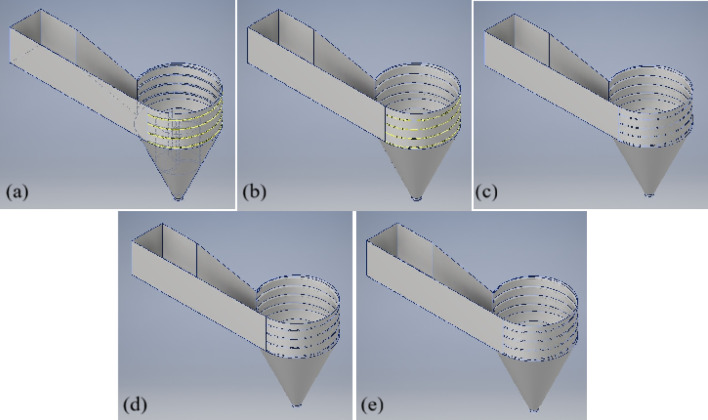
Varying ducted airfoils of NACA0012, NACA0015, NACA0018, NACA0021, and NACA0024 for (**a**), (**b**), (**c**), (**d**), and (**e**) respectively.

Finally, ducted airfoils were varied as follows: NACA0012, NACA0015, NACA0018, NACA0021, and NACA0024, as shown in Fig. 4.

The selection of the design parameters investigated was guided by initial numerical observations and practical considerations. The range of airfoil axial spacing was chosen to study the influence of flow guidance on vortex development; smaller spacing values were not extended further once the results showed marginal variation, while larger spacing values were used to assess their effect on swirl attenuation. For the selected airfoil chord sizes, very small chord sizes resulted in significantly increased mesh requirements and computational cost, as well as being unsuitable for future fabrication. For larger chord sizes, the numerical results exhibited stable trends, justifying the selected upper limits.

Regarding airfoil profile selection, the primary objective was to investigate the influence of airfoil thickness on vortex formation and rotor performance. Symmetric NACA four-digit airfoils with varying thickness ratios were used to isolate thickness effects. Throughout all simulations, the basin geometry was kept fixed to ensure that performance variations are a result of modified basin wall parameters. The runner was positioned at the basin center and consisted of three blades with an outer diameter of 40 mm, connected to a vertical shaft of 20 mm diameter. The basin geometry included a top chamber diameter of 400 mm, a bottom outlet diameter of 60 mm, and a total basin height of 610 mm.

**Fig. 5:**
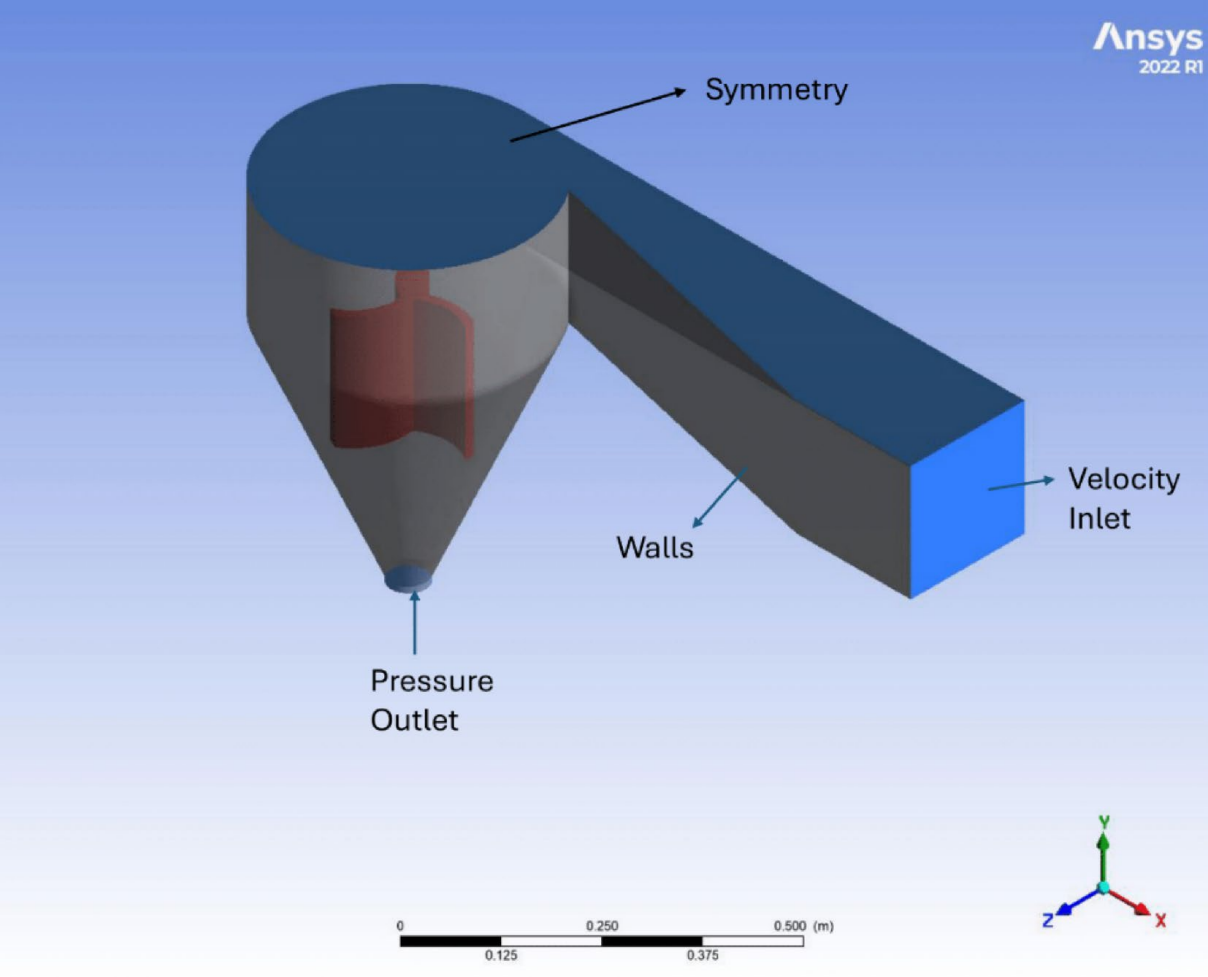
Simulation boundary conditions.

The inlet was defined as a uniform velocity inlet with a magnitude of 1.6 m/s, while the outlet was specified as a pressure outlet at atmospheric pressure. All solid walls, including basin surfaces and turbine blades, were modeled with no-slip boundary conditions as shown in Fig. 5. The Multiple Reference Frame (MRF) approach was used to model rotor rotation at 65 RPM under steady-state conditions, with pressure–velocity coupling handled using the SIMPLE algorithm.

A steady-state solver coupled with the Multiple Reference Frame (MRF) approach was employed to predict the time-averaged hydrodynamic performance of the turbine. Although gravitational water vortex flows are inherently unsteady, the primary performance indicators considered in this study represent time-average quantities. The steady-state solver greatly reduces computational cost while maintaining a parametric study of basin geometry and turbine configuration^23^.

To quantitatively evaluate the strength of the vortex generated within the basin and to support the analysis of flow performance, the swirl intensity was assessed using the dimensionless swirl number *S*. The swirl number was calculated on a horizontal plane located at the runner mid-height using Eq. 1^24^.

1 $$\:\begin{array}{c}S=\frac{\rho\:\:{u}_{x}\:{u}_{\theta\:}\:{r}^{2}}{R\:\rho\:\:{{u}_{x}}^{2}\:r}\:\end{array}$$

Where *u*_*x*_ is the axial velocity (m/s), *u*_*θ*_ is the tangential velocity (m/s), *r* is the radial position (m), and *R* is the basin radius (m).

To carry out a non-dimensional performance assessment and comparison between different operating conditions, the turbine performance was also evaluated using the tip speed ratio (TSR) which is used to characterize the operating scheme and efficiency of hydrokinetic turbines. In this study, the TSR (λ) was calculated using the runner rotational speed (ω), rotor radius (R), and inlet current velocity (U), as shown in Eq. 2.

2 $$\:\begin{array}{c}\lambda\:=\frac{\omega\:\:R}{U}\end{array}$$

### Grid independence verification

**Fig. 6:**
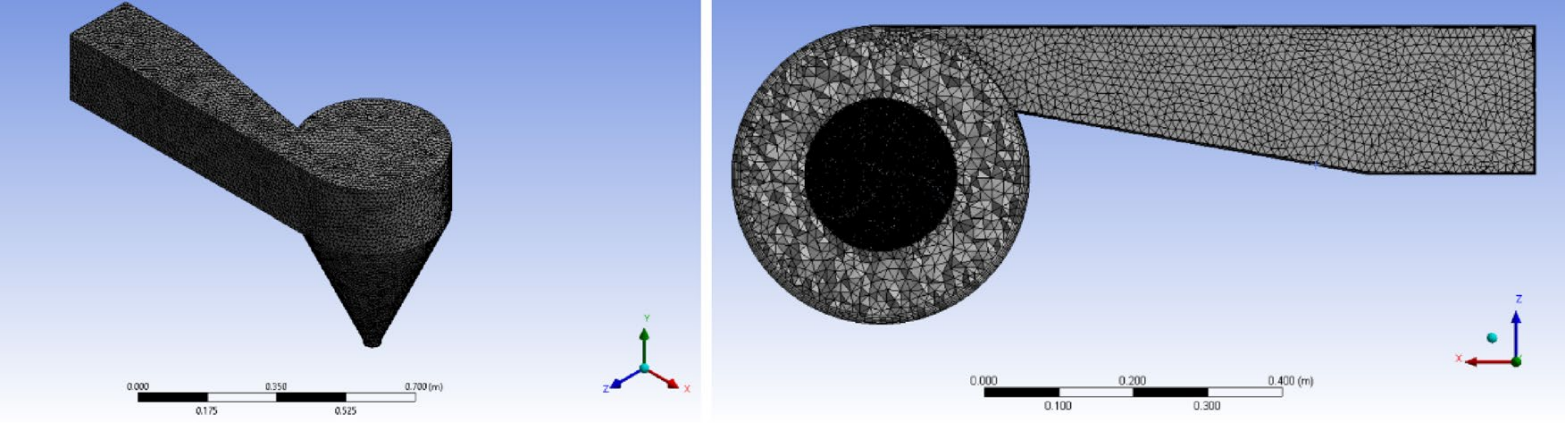
Different sections of the model mesh with the airfoil on the basin wall.

The computational domain was developed using ANSYS Meshing, employing an unstructured tetrahedral mesh with localized refinement in regions of high velocity gradients, particularly near the basin wall airfoils and turbine blades as shown in Fig. 6 with a minimum edge size of 0.1 mm, a face size of 0.23 mm, and a maximum size of 0.5 mm. Inflation layers were applied around the blades to accurately capture near-wall flow behavior with a first layer thickness of 0.1 mm.

**Fig. 7:**
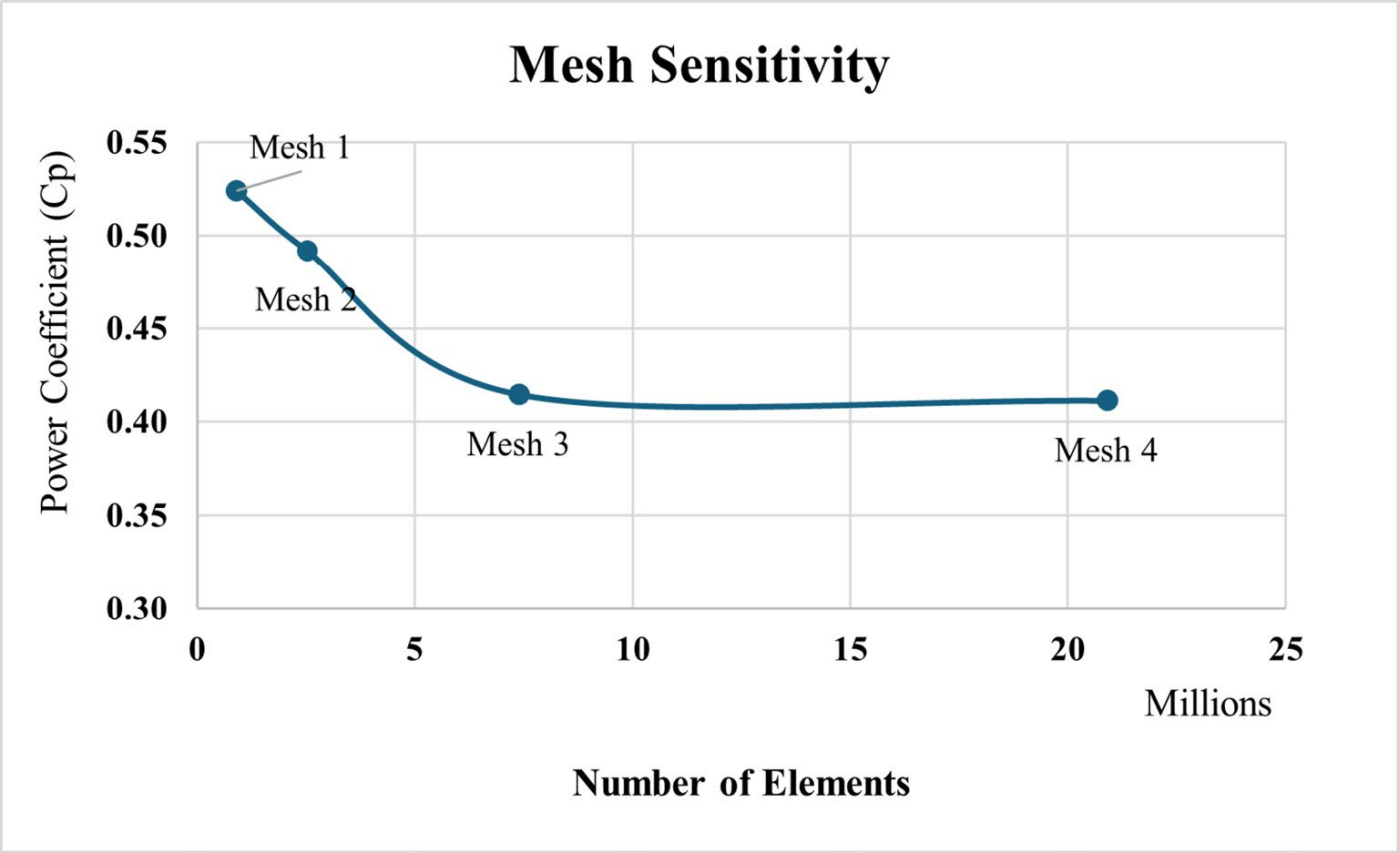
Mesh sensitivity study for numerical simulations.

A grid independence study was conducted to ensure that the numerical results were not affected by mesh resolution. Four different mesh densities were generated by using a refinement ratio of √2. The power coefficient (*Cp*) was selected as the primary convergence indicator. As shown in Fig. 7, the difference in *C*_*p*_ between Mesh 3 and Mesh 4 was less than 1% (0.415 to 0.411), demonstrating that further mesh refinement produced negligible changes in results. Thus, Mesh 3, consisting of approximately 20 million elements, was selected for all subsequent simulations.

### Numerical model validation

The SST k–ω turbulence model was employed in this study due to its proven capability in resolving adverse pressure gradients and near-wall effects which are dominant in gravitational water vortex systems. The model has been previously validated against experimental measurements for similar vortex-based hydropower configurations by^23^, demonstrating good agreement in velocity distribution and vortex structure. In the present study, the same turbulence model was adopted based on a numerical validation using the R10 runner geometry of Bajracharya et al.^23^, where maximum *C*_*p*_ was 0.3113.

The current model’s output power (P_out_) was calculated using Eq. 3, the angular velocity (ω) of the runner, and the torque (T) obtained from the simulation at maximum C_p_.

3 $$\:\begin{array}{c}{P}_{out}=T\times\:\omega\:=\frac{11.56\times\:65\times\:2\pi\:}{60}=78.6932\:W\end{array}$$

The current model’s input power (P_in_) was calculated using Eq. 4, water density (ρ), water flowrate (Q), gravitational acceleration (g), and water head (h).

4 $$\:\begin{array}{c}{P}_{in}=\rho\:Qgh=1\times\:1.6\times\:2\times\:2\times\:9.81\times\:4=251.136\:W\end{array}$$

Finally, the current model’s *C*_*p*_ was calculated using Eq. 5 and compared to the validation model’s *C*_*p*_.

5 $$\:\begin{array}{c}{C}_{p}=\frac{{P}_{out}}{{P}_{in}}=\frac{78.6932}{251.136}=0.313\end{array}$$

The validation results show error margins of 0.543% between the experimental model of Bajracharya et al.^23^, and the current numerical model^25^ for maximum and optimum C_p_ values respectively, as compared to 36.91% and 13.33% errors between Bajracharya et al.'s [23] experimental and computational models at maximum and optimum C_p_ values respectively.

This provides confidence in the reliability of the SST k–ω model for predicting vortex behavior and turbine hydrodynamic performance in the current configuration as it provides the closest match to experimental vortex profiles in gravitational water vortex turbines^26^.

## Results and discussion

**Fig. 8:**
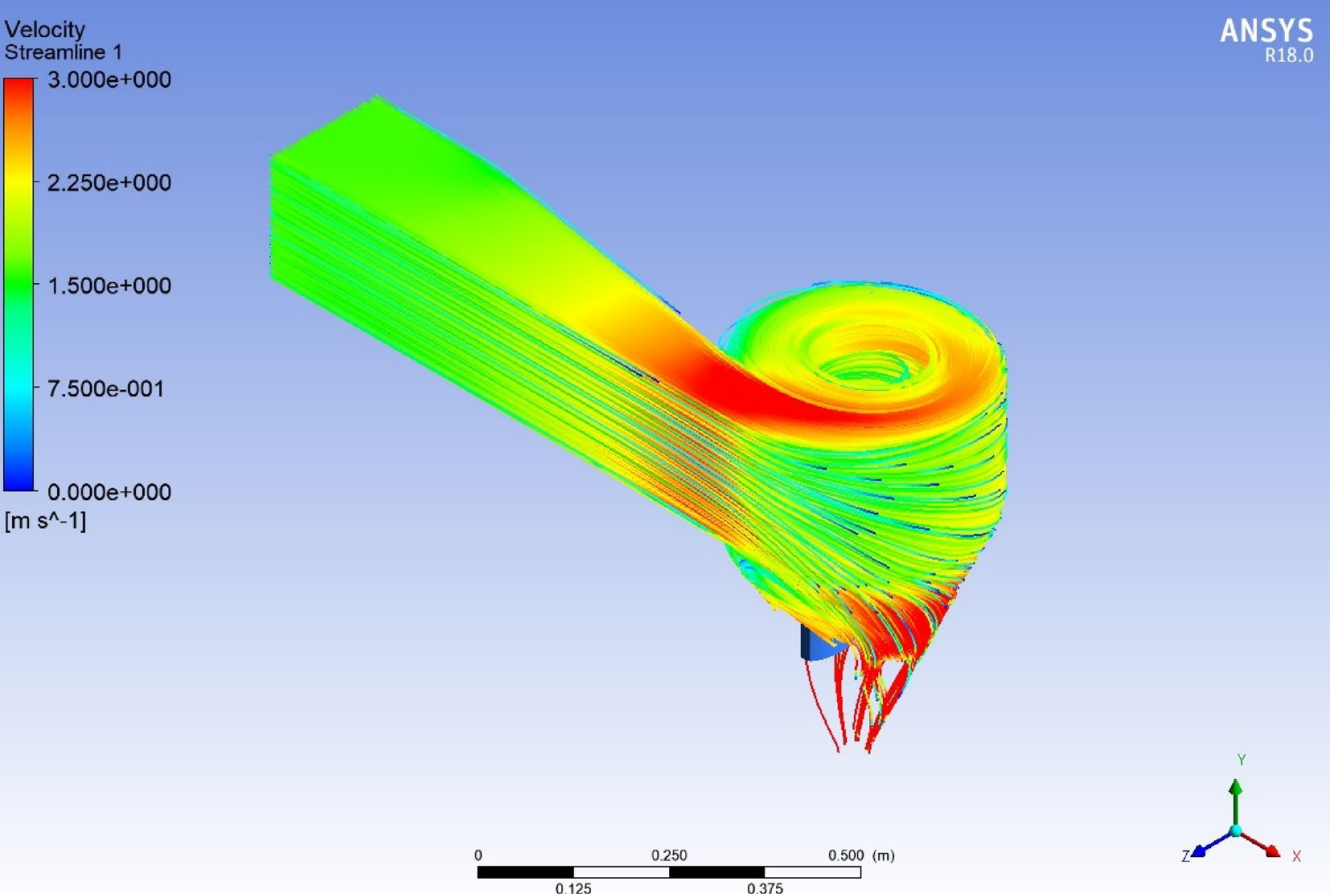
Velocity streamlines of the baseline basin.

The numerical results presented in this section analyze the hydrodynamic behavior and performance of the runner operating within the modified basin configurations. All simulations were initially conducted at a constant rotational speed of 65 RPM and an inlet current velocity of 1.6 m/s to establish a reference condition shown in Fig. 8. The influence of basin wall modifications on turbine performance is evaluated through variations in flow, velocity distribution, and power coefficient.

The flow is characterized by the formation of a strong swirling motion, with maximum tangential velocities near the basin wall as a result of centrifugal forces. Simultaneously, the rotating flow creates a low-pressure region near the basin center, generating a suction effect that guides the flow toward the outlet. The V-shaped basin geometry strengthens this behavior by gravitational acceleration of the flow toward the bottom orifice.

### Effect of airfoil axial spacing on rotor performance

**Fig. 9:**
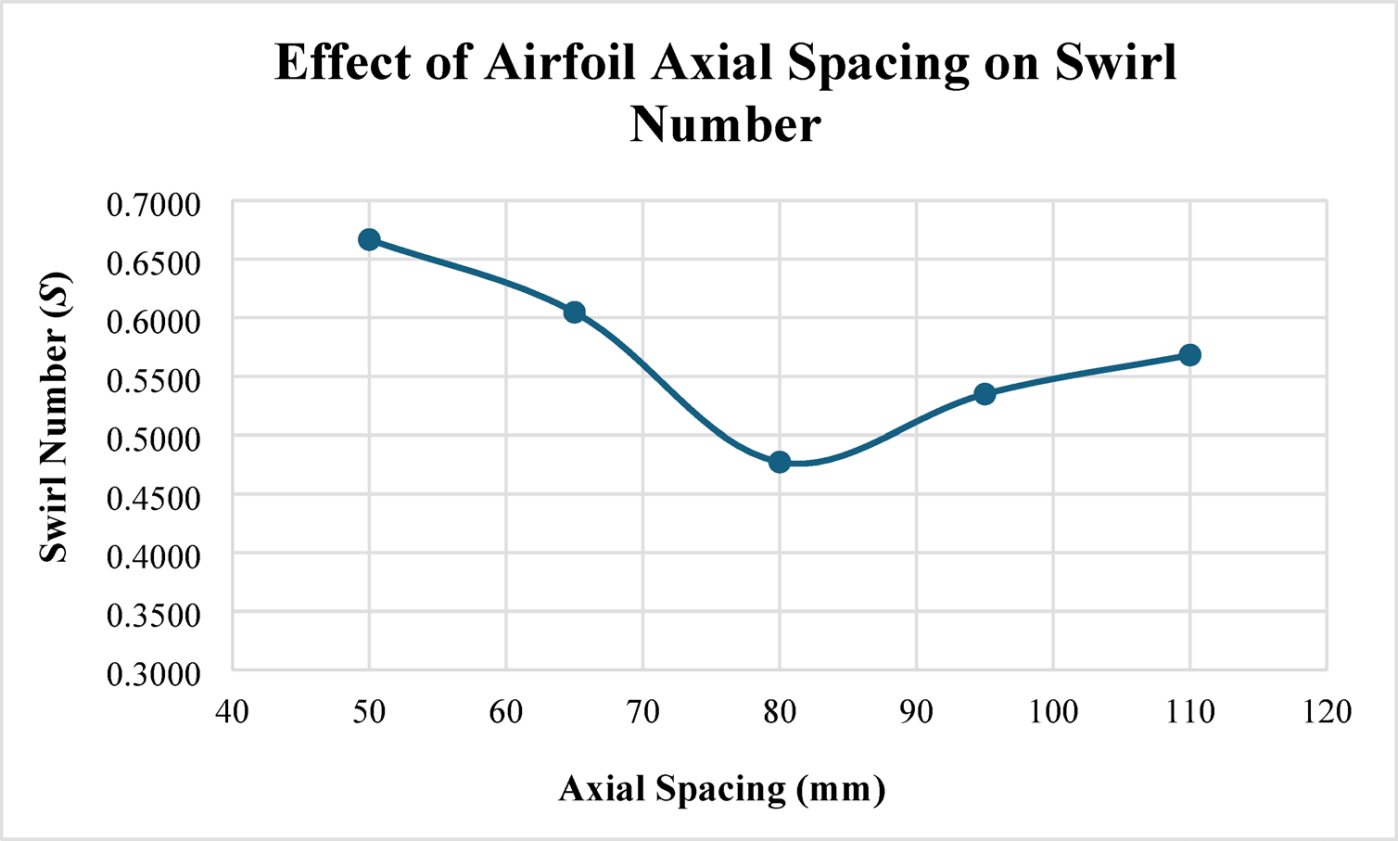
Swirl number variation for basin wall airfoil axial spacing.

**Fig. 10:**
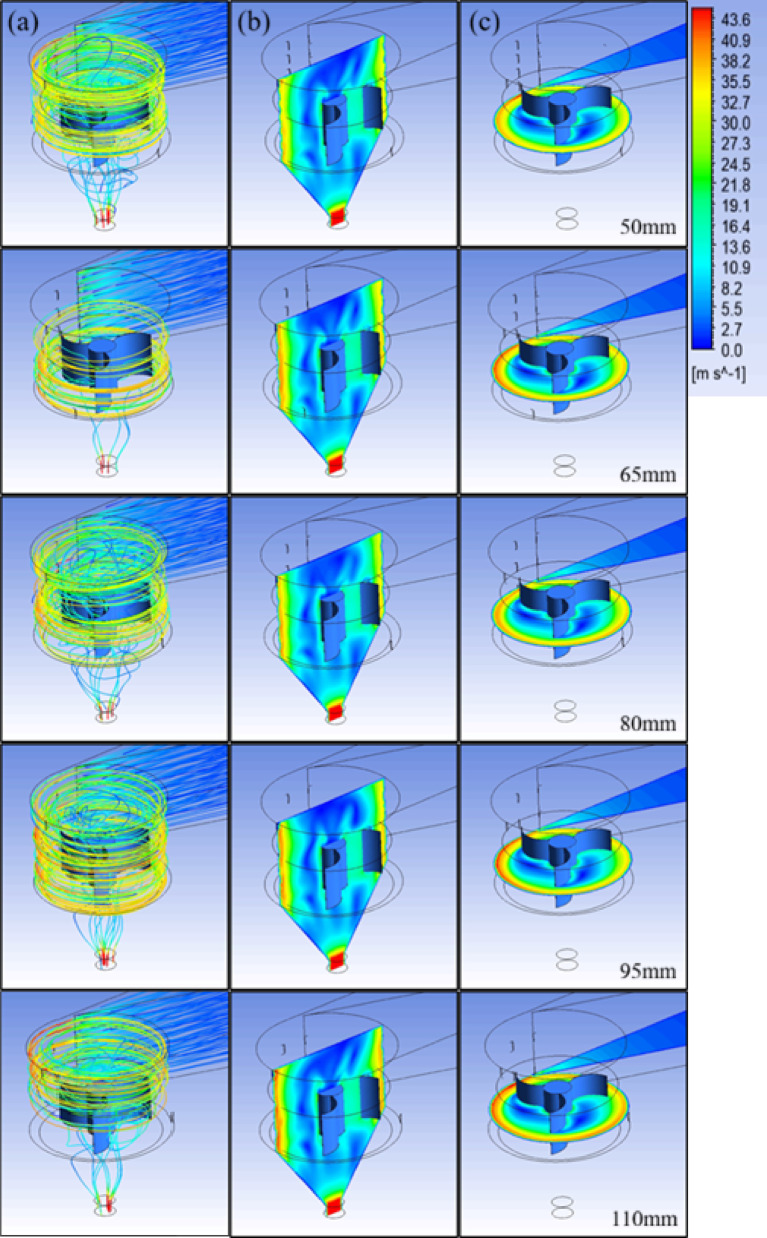
Streamlines (**a**) and velocity contours (**b**) and (**c**) of the runner inside the V-shaped basin for airfoil’s axial spacings of 50, 65, 80, 95, and 110 mm respectively.

The influence of airfoil axial spacing along the basin wall was first studied to evaluate its effect on flow structure and rotor performance as illustrated in Fig. 9. Introducing helical ducted airfoils along the basin wall greatly affected the interaction between the swirling flow and the runner. As illustrated by the streamlines and velocity contours in Fig. 10, modifying the axial spacing directly affected both the tangential and vertical velocity components around the rotor. The strongest swirl intensities were obtained for the smallest axial spacing of 50 mm, followed by 65 mm, 110 mm, and 95 mm, respectively, while the weakest swirl was observed at an axial spacing of 80 mm as summarized in Fig. 9. In all cases, the maximum tangential velocities were concentrated near the basin wall due to centrifugal forces, after which the flow followed a helical path toward the bottom outlet. Increasing the axial spacing led to an increase in the vertical velocity component, indicating that wider spacing achieves downward acceleration of the flow with weak swirl near the rotor.

**Fig. 11:**
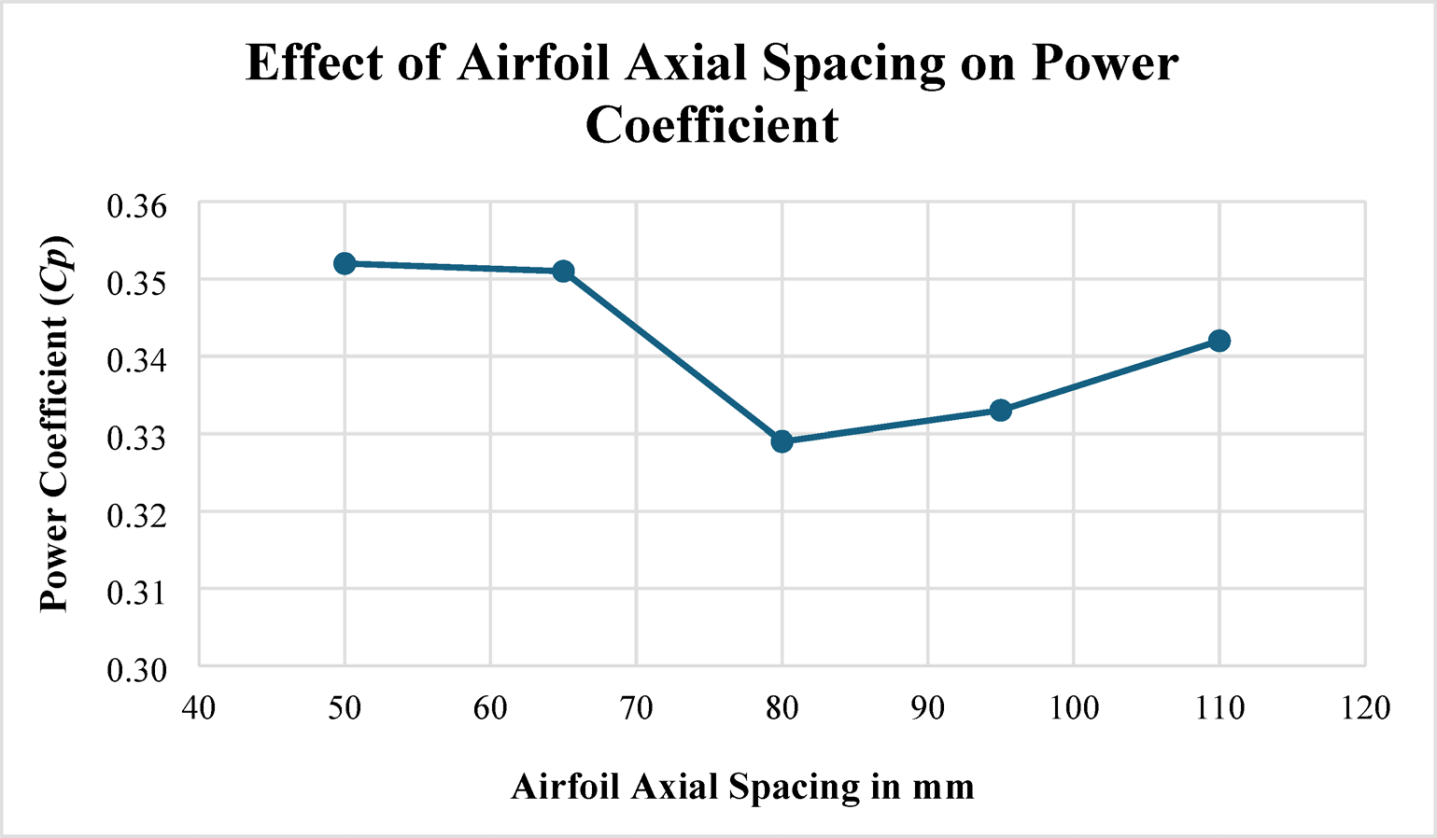
Power coefficients of the runner affected by the basin wall airfoil axial spacing.

The impact of axial spacing on rotor performance is expressed through the power coefficient values summarized in Table 1 and illustrated in Fig. 11. Compared to the baseline basin configuration without wall modifications at an optimum *C*_*p*_ of 0.313, all axial spacing cases resulted in improved performance. The maximum power coefficient of 0.352 was achieved at an axial spacing of 50 mm, corresponding to a 12.5% improvement, while the minimum value of 0.329 occurred at 80 mm, representing a 5.1% increase over the baseline.

**Table 1:** The power coefficient of the runner with respect to different cases.

Parameter		Power coefficient	Percentage (%)
Spacing	50 mm	0.352	12.5
	65 mm	0.351	12.1
	80 mm	0.329	5.1
	95 mm	0.333	6.4
	110 mm	0.342	9.3
Sizing	35 mm	0.419	33.9
	50 mm	0.340	8.6
	65 mm	0.353	12.8
	80 mm	0.313	0.2
	95 mm	0.317	1.3
Airfoil	NACA0012	0.366	16.9
	NACA0015	0.333	6.4
	NACA0018	0.337	7.7
	NACA0021	0.359	14.7
	NACA0024	0.379	21.1

The results show how the airfoil axial spacing directly influences the balance between tangential and axial velocity components at the runner location. Small axial spacing enhances tangential momentum reaching the rotor blades by increasing the swirl near the basin wall which increases the effective torque acting on the rotor. However, smaller spacing can trap the flow, increasing vertical acceleration and changing blade momentum. On the other hand, larger axial spacing weakens flow paths, resulting in reduced tangential velocity and lower torque. The observed variation in power coefficients reflects an exchange between swirl enhancement and flow uniformity at the rotor.

### Effect of airfoil chord sizing on rotor performance

**Fig. 12:**
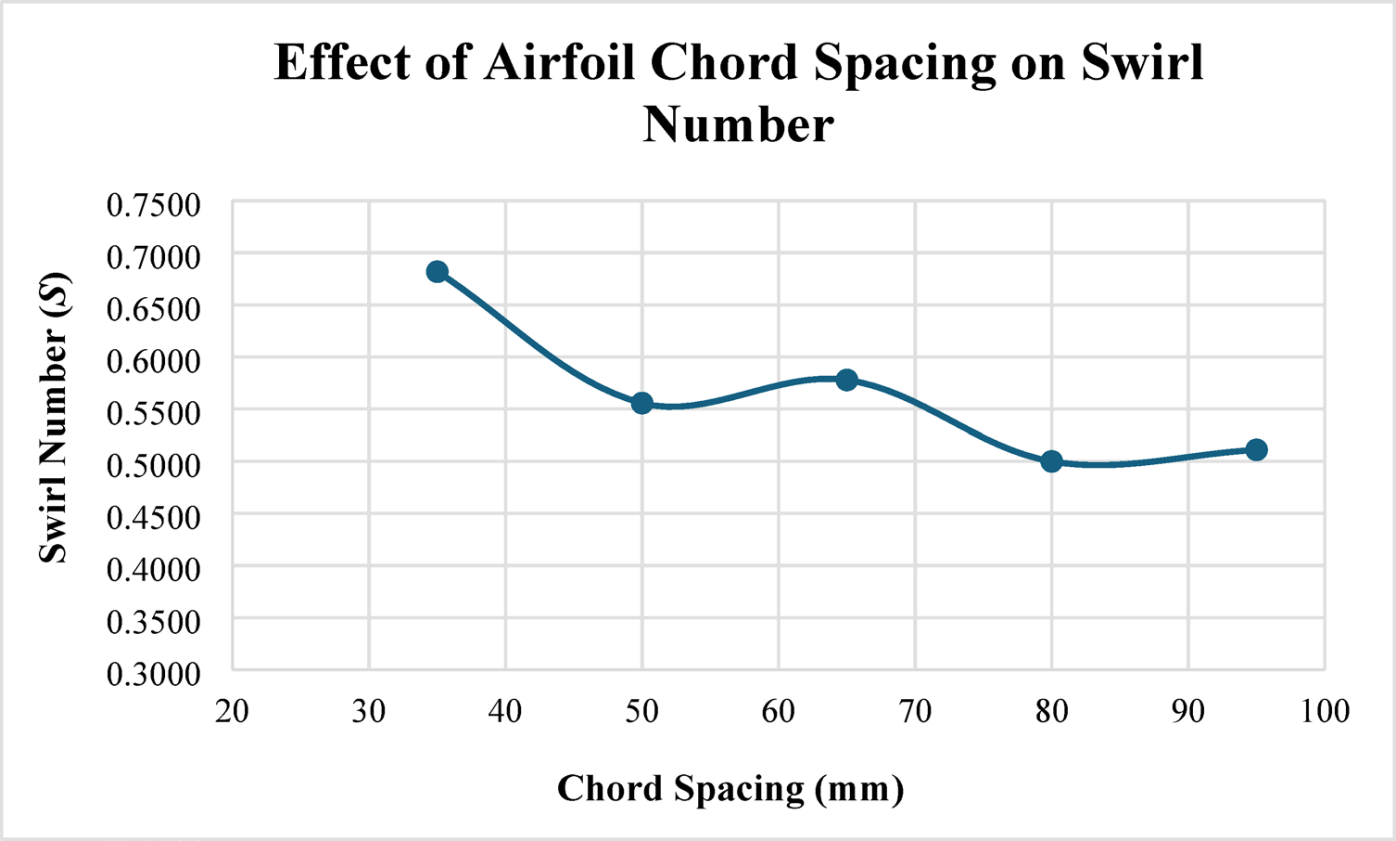
Swirl number variation for basin wall airfoil chord spacing.

**Fig. 13:**
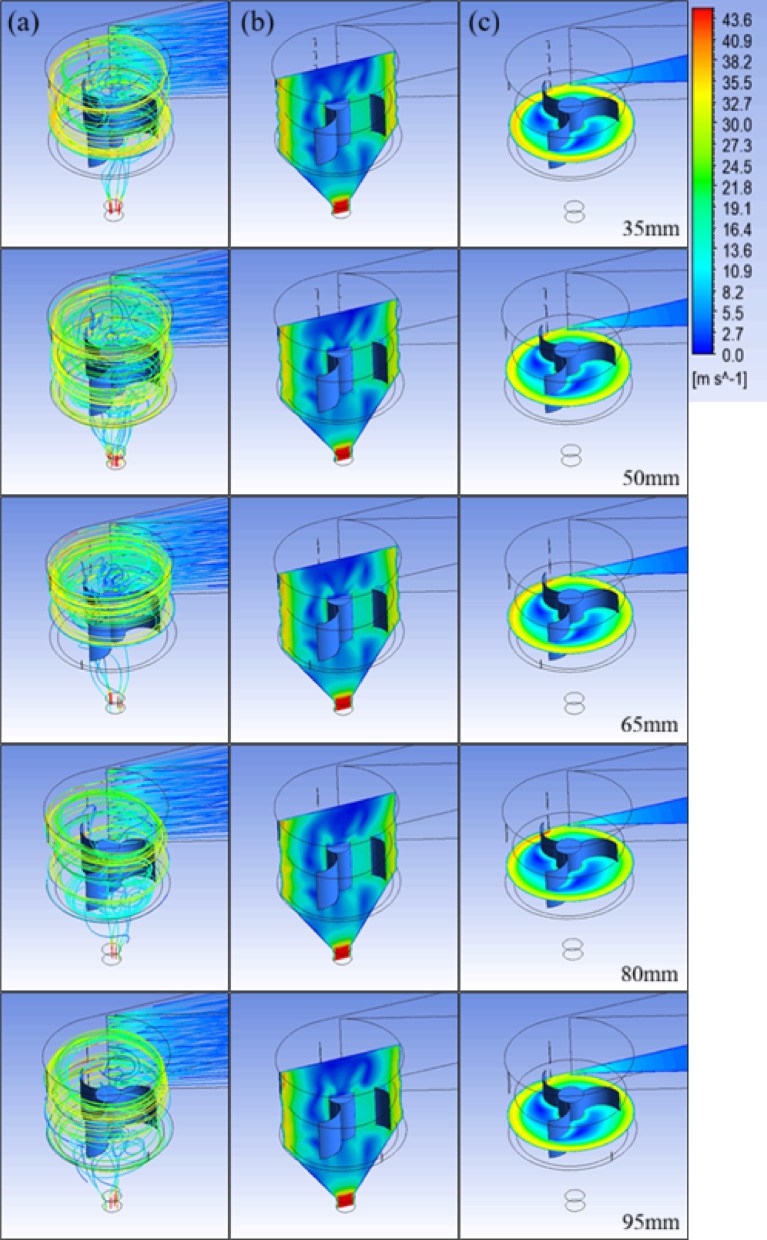
Streamlines (**a**) and velocity contours (**b**) and (**c**) of the runner inside the V-shaped basin of the ducted airfoil with a sizing of 35 mm, 50 mm, 65 mm, 80 mm, and 95 mm respectively.

The influence of ducted airfoil chord sizing was examined to assess its effect on flow structure and rotor performance as shown in Fig. 12. Varying the chord sizing changes the balance between flow guidance and blockage within the basin, thereby changing the velocity distribution up to the runner. The resulting changes in flow behavior are illustrated by the streamlines and velocity contours in Fig. 13. The smallest chord size of 35 mm generated the strongest swirl intensity near the basin wall, while larger chord sizes led to a reduction in tangential velocity magnitude. As summarized in Table 1, the swirl velocity decreased as the chord size increased from 35 mm to 95 mm, with the weakest swirl observed at larger chord sizes as summarized in Fig. 12. At the same time, increasing the chord size resulted in a reduction of the vertical velocity component, indicating that larger airfoils restrict downward flow by increasing flow resistance within the basin.

**Fig. 14:**
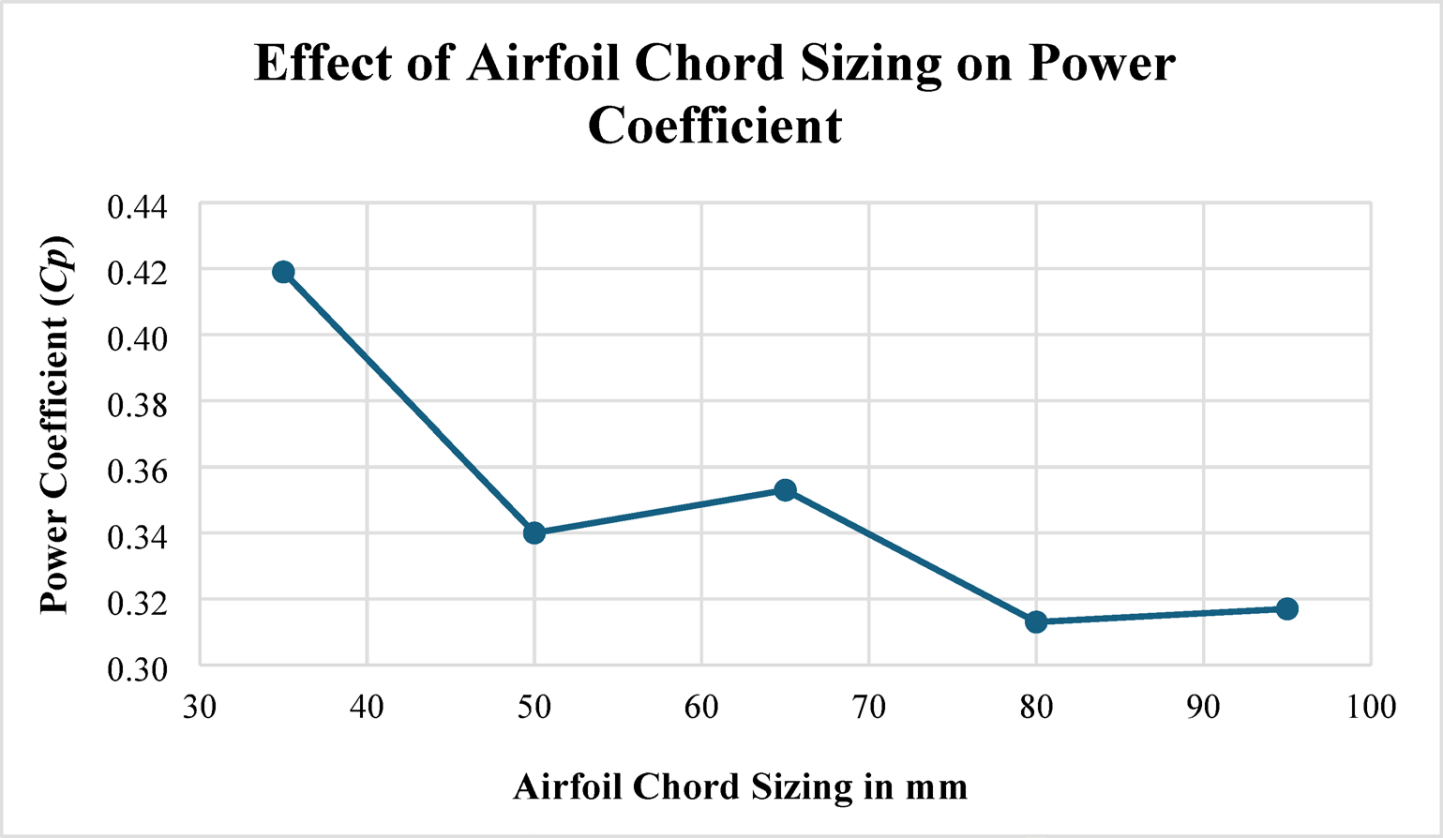
Power coefficients of the runner as affected by the basin wall airfoil sizing.

The impact of chord size on rotor performance is reflected in the power coefficient trends shown in Fig. 14. The highest power coefficient of 0.419 was obtained from the smallest chord size of 35 mm, corresponding to a 34.4% improvement over the baseline basin configuration. In contrast, the lowest power coefficient of 0.313 occurred at a sizing of 80 mm, which nearly matches the baseline performance. These results demonstrate that larger chord sizes reduce the effectiveness of flow guidance despite high velocities near the basin wall.

The airfoil chord sizing affects rotor performance by controlling flow passage and swirl consistency before the flow reaches the runner. Smaller sizing reduces flow obstruction but provides enough guidance to increase tangential velocity which results in higher torque on the rotor blades. As the sizing increases, flow blockage increases and reduces the tangential velocity on the rotor which is shown by the reduction in power coefficient even with high velocity near the basin wall.

### Effect of helical airfoil profile (NACA series) on rotor performance

**Fig. 15:**
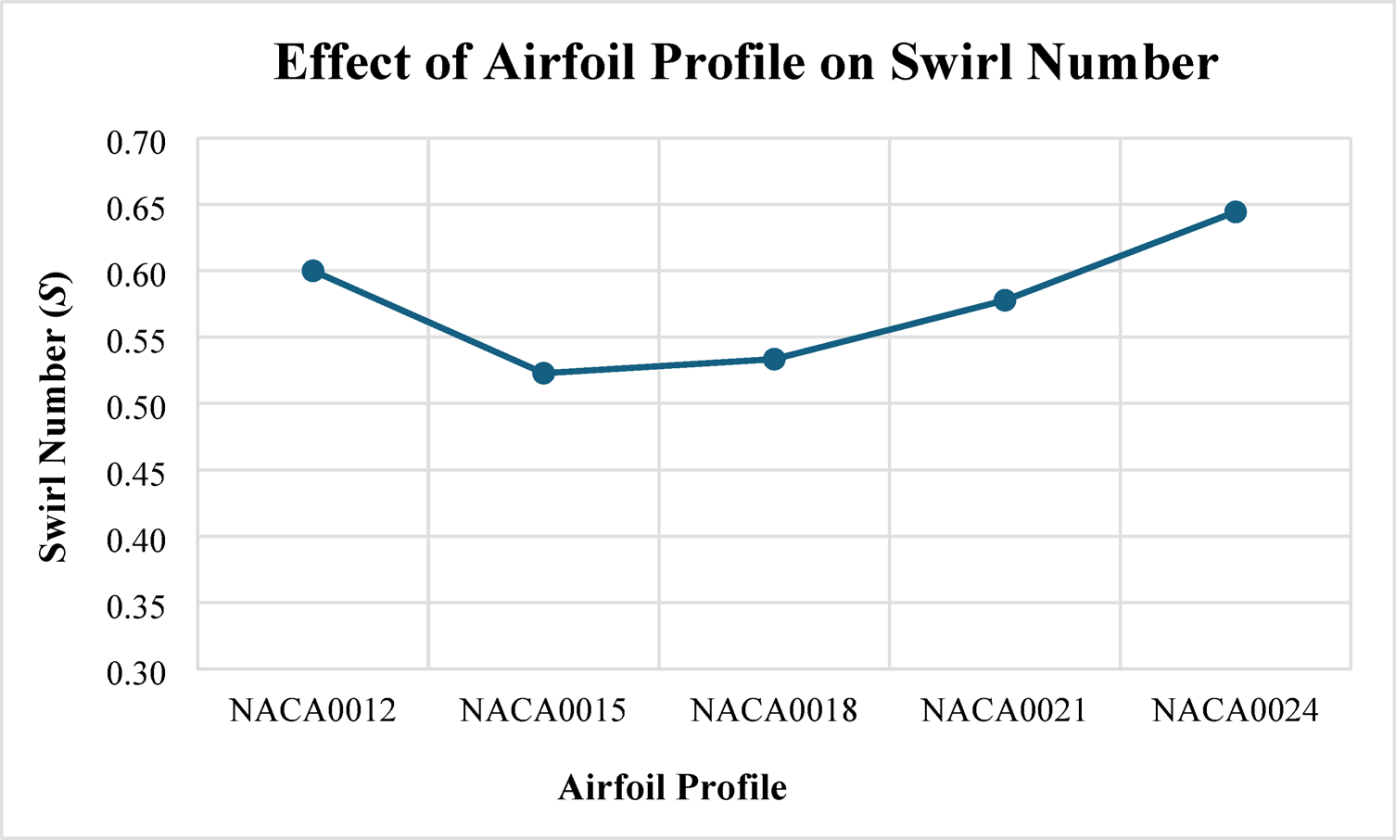
Swirl number variation for airfoil profile.

**Fig. 16:**
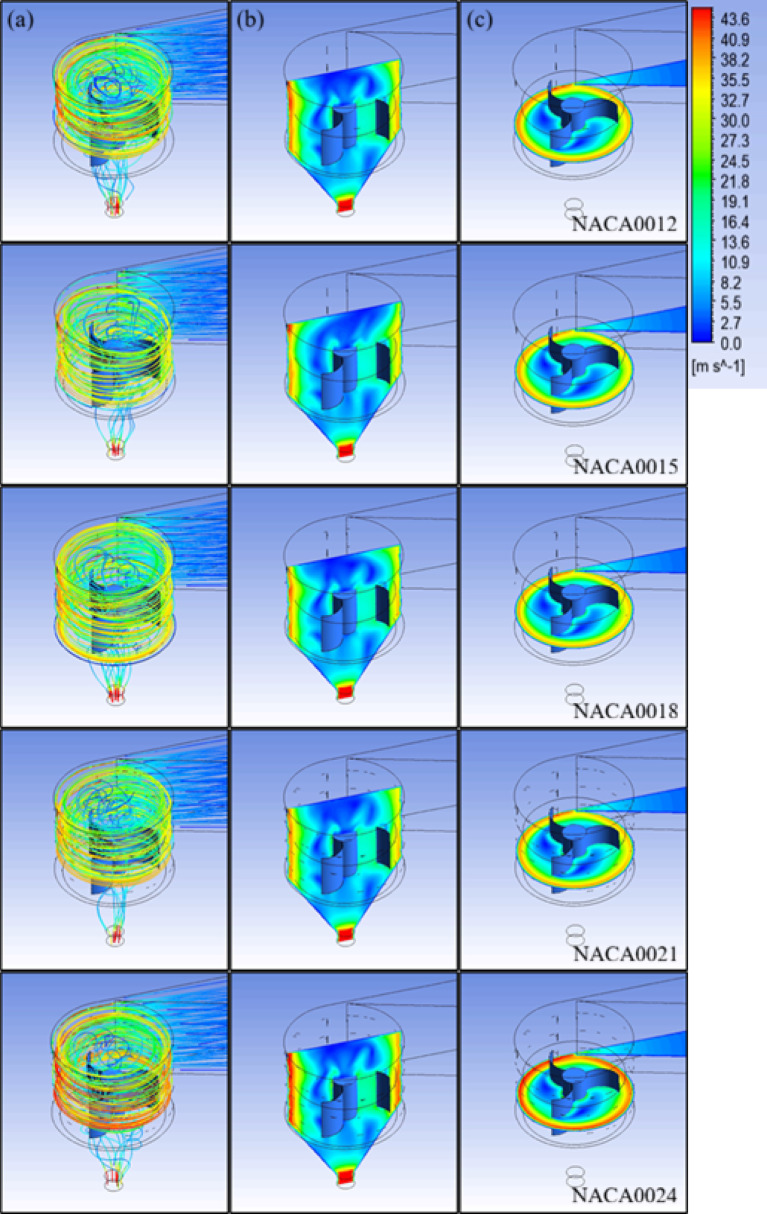
Streamlines (**a**) and velocity contours (**b**) and (**c**) of the runner inside the V-shaped basin of the ducted airfoils of NACA0012, NACA0015, NACA0018, NACA0021, and NACA0024 respectively.

The effect of helical airfoil profile thickness was investigated using symmetric NACA four-digit airfoils (NACA0012, NACA0015, NACA0018, NACA0021, and NACA0024). Varying the airfoil thickness modified the flow guidance characteristics along the basin wall, which in turn changes the velocity distribution and swirl intensity reaching the runner. The resulting flow patterns are illustrated by the streamlines and velocity contours in Fig. 15. An increase in airfoil thickness improved the vertical and tangential velocity components within the basin. Among the proposed profiles, the NACA0024 airfoil produced the highest swirl intensity near the basin wall, followed by NACA0012, NACA0021, and NACA0018, while the weakest swirl was observed for NACA0015, as summarized in Table 1; Fig. 16. Thicker airfoils created stronger flow turning and more stable swirling, whereas thinner airfoils provided weaker guidance, resulting in reduced swirling flow uniformity approaching the rotor.

**Fig. 17:**
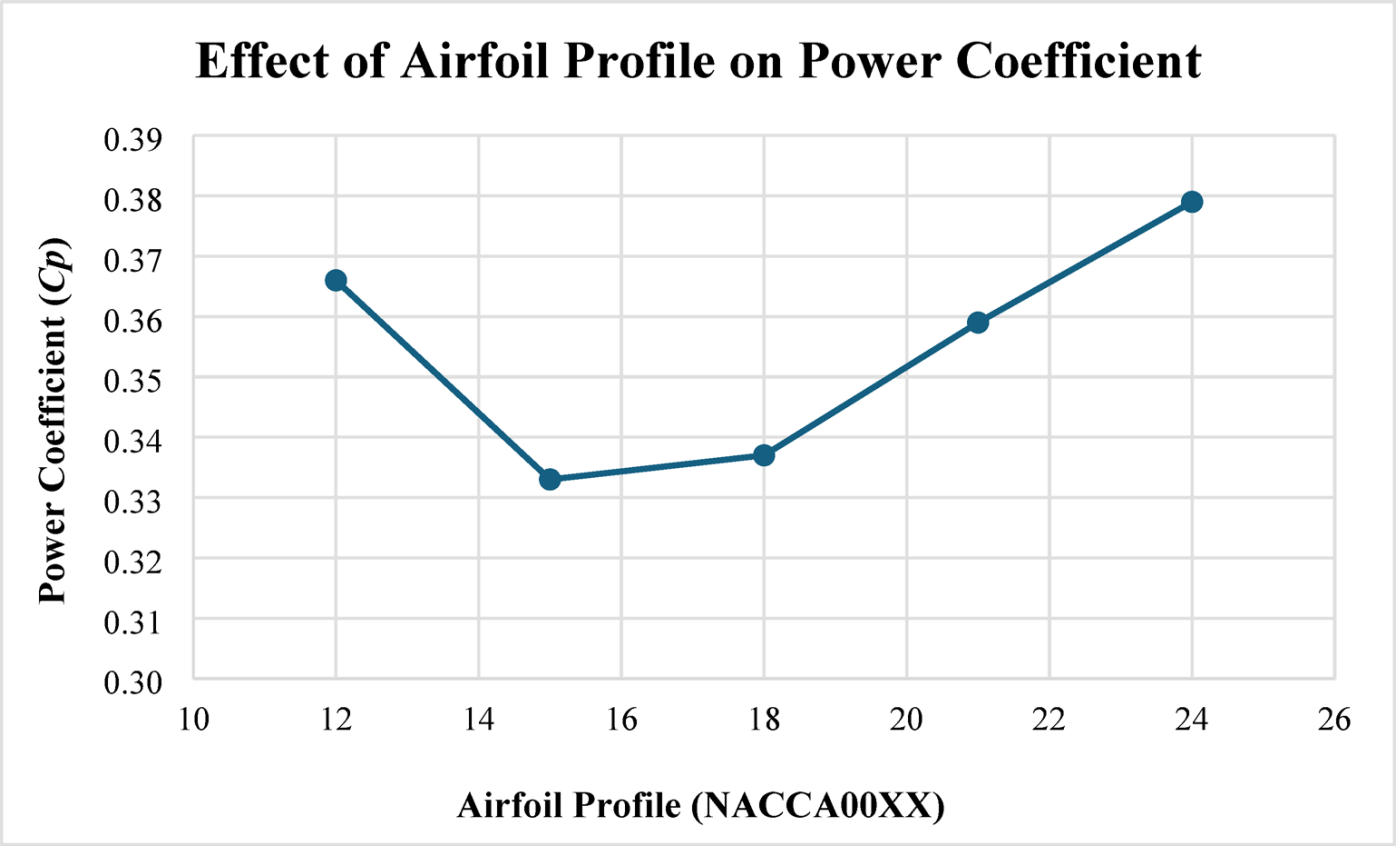
Power coefficients of the runner affected by the basin wall airfoil.

These flow modifications’ effects are shown in the rotor performance trends in Fig. 17. The highest power coefficient of 0.379 was achieved using the NACA0024 airfoil, corresponding to a 21.4% improvement relative to the baseline configuration. In contrast, the lowest power coefficient of 0.333 was obtained for the NACA0015 airfoil, indicating little improvement in tangential momentum transfer to the rotor. These results show that airfoil thickness plays a critical role in the effectiveness of basin-induced flow control.

The airfoil thickness affects rotor performance by changing the strength of the swirl entering the runner region. Thicker airfoils improve the swirl and reduce flow separation on the basin wall which increases the tangential velocity on the rotor radius and improves blade loading. This results in higher torque and power coefficient as shown for the NACA0024 airfoil. In contrast, thinner airfoils reduce flow guidance and subsequently tangential momentum transfer which lowers rotor efficiency.

From a hydrodynamic perspective, the better performance of the thicker NACA0024 airfoil can be linked to its enhanced flow-guiding ability and improved stability of the induced vortex. The increased thickness provides a stronger flow turning along the basin wall and reduces flow separation. This results in a more coherent swirling structure with a higher tangential velocity component at the rotor radius. Although lift-to-drag characteristics are not explicitly calculated in this study, the observed flow behavior suggests that thicker airfoils achieve more balance between flow guidance (lift-like effect) and viscous losses (drag-like effect), thereby improving torque generation and power coefficient. In contrast, thinner airfoils create weaker flow turning and are subject to boundary-layer separation along the basin wall, leading to reduced swirl and tangential momentum transfer to the rotor.

### Parameter optimization and operating range

Parametric optimization was conducted to identify the most effective combination of basin wall airfoil parameters under varying operating conditions. Five inlet current velocities (1.6, 2.0, 2.4, 2.8, and 3.1 m/s) were used to assess rotor performance across a range of flow conditions. Based on the results, the two best configurations were selected for each design variable: airfoil profile (NACA0024 and NACA0012), airfoil chord sizing (35 mm and 65 mm), and airfoil axial spacing (50 mm and 65 mm). This resulted in eight combined configurations, which were simulated and compared, as summarized in Table 2.

The results in Table 2 indicate that the optimized configurations show better results compared to the baseline basin across all inlet velocities. Among the tested combinations, the configuration using NACA0024, 35 mm chord size, and 50 mm axial spacing achieved the highest power coefficients for all operating conditions. At an inlet velocity of 3.1 m/s, this configuration reached a maximum power coefficient of 0.736, while maintaining better performance even at lower velocities. In contrast, configurations incorporating larger chord sizes or wider axial spacing showed reduced power coefficients.

**Table 2:** Power coefficients of the runner for different cases with 5 current velocities.

Cases				Power Coefficients				
#	Airfoil	Spacing (mm)	Sizing (mm)	1.6 m/s	2 m/s	2.4 m/s	2.8 m/s	3.1 m/s
1	NACA0012	50	35	0.330	0.425	0.504	0.562	0.620
2		50	65	0.328	0.411	0.494	0.544	0.645
3		65	35	0.321	0.423	0.484	0.574	0.612
4		65	65	0.330	0.442	0.514	0.594	0.658
5	NACA0024	50	35	0.355	0.470	0.571	0.684	0.736
6		50	65	0.347	0.440	0.556	0.644	0.716
7		65	35	0.345	0.450	0.555	0.674	0.706
8		65	65	0.344	0.433	0.536	0.624	0.704

**Fig. 18:**
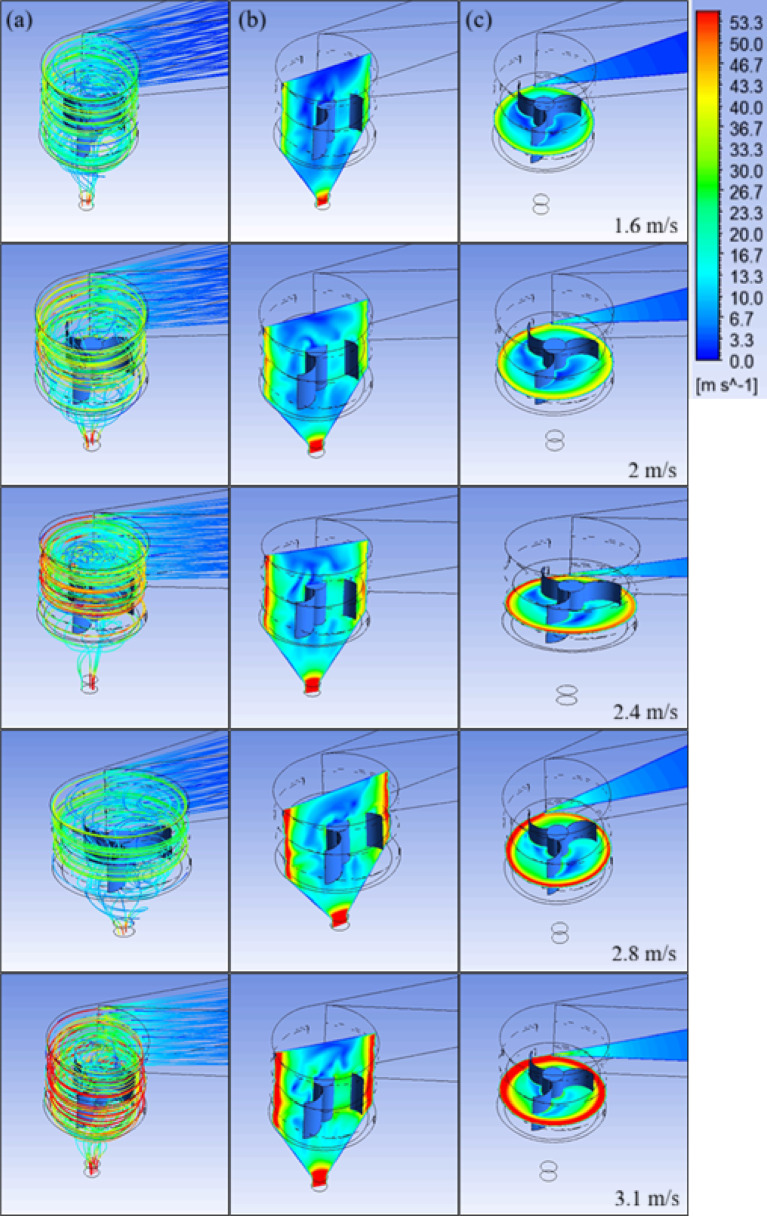
Streamlines (**a**) and velocity contours (**b**) and (**c**) of the runner inside the basin of the ducted airfoils with NACA0024, sizing of 35 mm, and spacing of 50 mm, with current velocities of 1.6, 2, 2.4, 2.8, and 3.1 m/s respectively.

The flow behavior of the optimum configuration is illustrated in Fig. 18, in streamlines and velocity contours for different inlet velocities. As the inlet velocity increased, the swirl intensity within the basin increased, with maximum tangential velocities concentrated near the basin wall and improved downward acceleration toward the outlet due to the effects of gravity and the V-shaped basin geometry. A low-pressure region developed near the basin center, creating a suction effect that maintained the vortex and improved rotor–flow interaction. The inlet velocity of 3.1 m/s generated the strongest vortex, followed by 2.8, 2.4, 2.0, and 1.6 m/s, respectively.

**Fig. 19:**
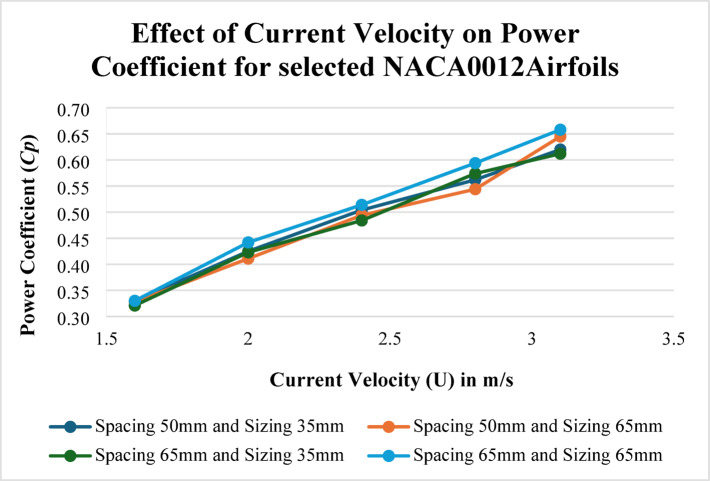
Power coefficients of the runner against different operating current velocities for the best NACA0012 cases used.

**Fig. 20:**
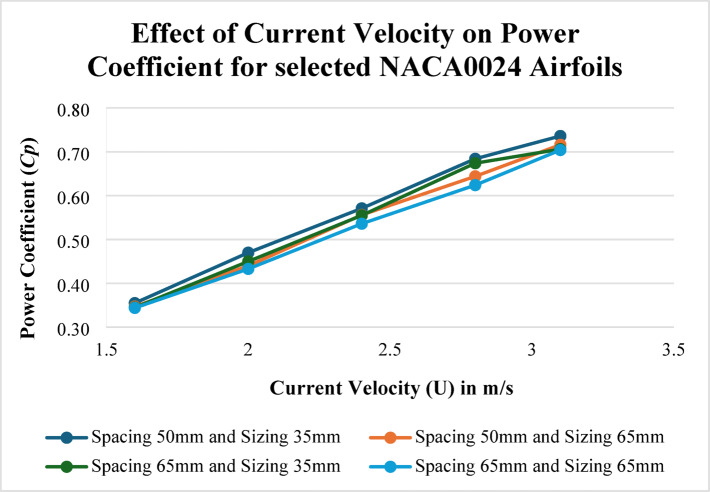
Power coefficients of the runner against different operating current velocities for the best NACA0024 cases used.

Across all cases, the optimized configurations produced higher power coefficients than the rotor operating in the unmodified basin, as shown in Figs. 19 and 20. This comparison confirms that basin wall modification using ducted airfoils significantly enhances rotor performance over a wide range of operating velocities. The improved performance of the optimal configuration is due to its ability to maximize tangential velocity at the rotor while minimizing vertical flow losses and non-uniform blade loading, thereby improving torque generation^27^.

The optimal configuration identified in this study is described as the condition that maximizes tangential velocity at the rotor while minimizing vertical flow losses and unequal blade loading, thus enhancing torque generation for the velocity range.

**Fig. 21:**
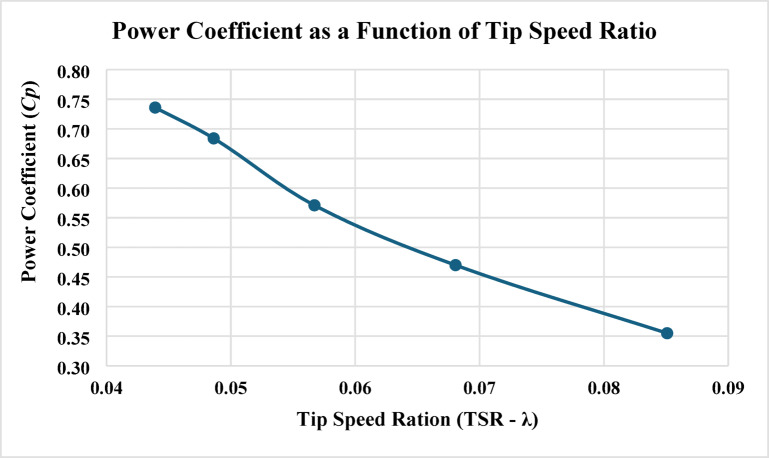
Variation of power coefficient (*C*_*p*_) with tip speed ratio (λ) for the optimized basin configuration (50 mm axial spacing, 35 mm airfoil sizing, and NACA0024 airfoil) at different inlet current velocities.

Figure 21 shows the change in power coefficient with (TSR) for the optimized cases. The results show a clear peak in *C*_*p*_ for low TSR thus indicating the optimum operating condition of the rotor. At higher TSR values, increased speed leads to higher flow losses and reduced blade loading whereas at lower TSR values, insufficient tangential momentum transfer reduces torque generation.

The findings of this study are consistent with trends found in previous literature on gravitational water vortex turbines but also extend prior findings through basin wall flow modification. Previous studies primarily focused on runner geometry and placement within cylindrical or conical basins, achieving power coefficients typically ranging between 0.25 and 0.50 depending on operating conditions and turbine configuration^13^. While other studies improved the efficiency using flow-guides such as baffle plates^15^. In comparison, the present study was able to reach higher power coefficients through the use of ducted airfoils mounted along the basin wall to improve tangential momentum transfer to the rotor. The obtained maximum power coefficient values fall within the upper range or exceed those reported in previous numerical studies, indicating the effectiveness of the proposed basin modifications.

Furthermore, while previous work focused on turbine-side optimization, the present results show that basin-induced flow control plays an equal role in rotor performance as observed in tip speed ratio trends and optimum operating range.

## Conclusion and future works

In conclusion, this study numerically investigated the performance enhancement of a gravitational water vortex hydrokinetic turbine for application in riverine systems through basin wall airfoil modifications. A computational fluid dynamics approach was used to evaluate the influence of airfoil axial spacing, chord sizing, and airfoil profile on flow behavior and turbine performance.

The results demonstrate that basin wall airfoil parameters have a significant impact on the generated vortex structure and rotor power coefficient. Among the cases, an airfoil axial spacing of 50 mm resulted in a power coefficient of 0.352, representing an improvement of approximately 12.5% compared to the baseline basin configuration. Reducing the airfoil chord size to 35 mm further enhanced turbine performance, yielding a maximum power coefficient of 0.419, corresponding to a 33.8% increase over the baseline case. In addition, increasing the airfoil thickness using the NACA0024 profile produced the highest performance among the tested airfoils, with a power coefficient of 0.379.

When combining the optimal parameters, the configuration consisting of a NACA0024 airfoil, 35 mm chord size, and 50 mm axial spacing consistently delivered the best performance across a wide range of inlet current velocities. The maximum power coefficient reached 0.736 at an inlet velocity of 3.1 m/s. Analysis based on tip speed ratio further indicated that the turbine operates optimally within a low TSR range (λ ≈ 0.044–0.057), which is characteristic of gravitational water vortex turbines operating under low-head, high-torque conditions.

Overall, the findings confirm that basin-induced flow control using ducted airfoils is an effective strategy for improving hydrokinetic turbine performance in riverine environments.

Future work will focus on extending the present numerical investigation to address the experimental validation of the optimized basin configuration which will be carried out using a laboratory prototype to verify the predicted power coefficients and vortex characteristics under controlled riverine flow conditions. In addition, transient and multiphase simulations will be conducted to capture unsteady vortex dynamics, free-surface fluctuations, and air–water interactions that cannot be fully resolved using steady-state single-phase modeling. Further hydrodynamic analysis will also be performed to quantify rotor-related performance indicators such as blade loading distribution, torque fluctuations, and effective lift–drag behavior within the vortex flow. Finally, future studies will explore manufacturability and long-term operational considerations to support the transition from numerical optimization to practical deployment of gravitational water vortex turbines in real riverine environments.

## Data Availability

The data used and/or analyzed in this study are available from the corresponding author upon reasonable request.

## Abbreviations

CFD: Computational fluid dynamics
MRF: Multiple reference frame
RPM: Revolution per minute
WHG: Whirlpool hydropower generator
Airfoil Axial Spacing: Center-to-center distance between successive ducted airfoils mounted along the basin wall
Ducted Airfoil: Aerodynamically shaped profile integrated within a surrounding duct and mounted along the basin wall to guide and enhance the flow entering the vortex chamber
TSR: Tip speed ratio
